# Effectiveness of dienogest in improving quality of life in Asian women with endometriosis (ENVISIOeN): interim results from a prospective cohort study under real-life clinical practice

**DOI:** 10.1186/s12905-019-0758-6

**Published:** 2019-05-16

**Authors:** Kitirat Techatraisak, Andon Hestiantoro, Soon Ruey, Maria Jesusa Banal-Silao, Mee-Ran Kim, Seok Ju Seong, Syarief Thaufik, Christiane Ahlers, So Young Shin, Byung Seok Lee

**Affiliations:** 1 Department of Obstetrics and Gynecology, Gynecologic Endocrinology Unit, Faculty of Medicine, Siriraj Hospital, Mahidol University, Bangkok, Thailand; 2 Department of Obstetrics and Gynecology, Faculty of Medicine, Cipto Mangunkusumo National Central General Hospital, Jakarta, Indonesia; 3 Department of Obstetrics and Gynecology, Sabah Women’s and Children’s Hospital, Kota Kinabalu, Malaysia; 4 Department of Obstetrics and Gynecology Reproductive Endocrinology and Fertility, St. Luke’s Medical Center, Philippine General Hospital, Metro Manila, Philippines; 5 Department of Obstetrics and Gynecology, The Catholic University Of Korea, Seoul St. Mary’s Hospital, Seoul, Korea; 6 Department of Obstetrics and Gynecology, CHA Gangnam Medical Center, CHA University, Seoul, Korea; 7 Department of Obstetrics and Gynecology, Hermina Pandanaran Hospital, Semarang, Indonesia; 8 Bayer Pharma AG, Wuppertal, Germany; 9 Former employee of Bayer AG, Seoul, Republic of Korea; 10 Division of Gynecologic Endocrinology and Infertility, Department of Obstetrics and Gynecology, Severance Hospital,Yonsei University, 50-1 Yonsei-ro, Seodaemun-gu, 120-752 Seoul, Republic of Korea

**Keywords:** Endometriosis, Health-related quality of life, Pain, Asian women, Clinical diagnosis

## Abstract

**Background:**

Dienogest has been shown to substantially improve endometriosis-associated symptoms such as debilitating chronic pelvic pain, and in turn, health-related quality of life (HRQoL). To date, there is no data on patient-reported outcomes reflecting the real-world practice in Asia where endometriosis is a relevant health, social and economic burden. This non-interventional, multi-center, prospective study aims to investigate the influence of dienogest on HRQoL.

**Methods:**

Asian women received dienogest (2 mg/daily) and were followed for 24 months. The effectiveness of dienogest to improve HRQoL and endometriosis-associated pelvic pain (EAPP) was assessed by patient-reported outcomes. HRQoL, especially the “pain” domain as primary endpoint, was evaluated with the Endometriosis Health Profile-30 (EHP-30) questionnaire. The numeric rating scale served to determine changes in the severity of EAPP. Within the presented interim analysis (data cut-off: 2017-11-27), the mean changes in EHP-30 and EAPP scores from baseline to 6 months upon availability of the data were evaluated. Treatment-emergent adverse events (TEAEs) and bleeding profiles were documented.

**Results:**

Dienogest therapy decreased EHP-30 scores in all assessed domains (score 0–100, lower scores indicate better HRQoL). Primarily, the “pain” domain was improved in 78.4% of patients. EAPP was reduced (score 0–10, lower scores reflect less pain), highlighted by a mean reduction of the pain score by − 4.5 points. Patients with a higher EAPP score at baseline had an increased response to dienogest (− 6.2 points mean change) compared to patients with low baseline EAPP severity (− 1.4 points mean change). Both surgically and clinically diagnosed patients described comparable pain reduction, as well as women with or without prior treatment.

Drug-related TEAEs were documented for 31.5% of patients, with amenorrhoea (5.9%) and metrorrhagia (5.1%) being the most common events. The bleeding pattern was changed upon dienogest, characterized by decreased normal bleeding (84.2 to 28.8%) and increased amenorrhea (3.2 to 42.9%) at 6 months.

**Conclusion:**

The data indicate an amelioration of HRQoL and EAPP upon dienogest therapy. No new safety signals were observed. Therefore, its use as first-line therapy for long-term management of debilitating and chronic endometriosis-associated pain represents an interesting option that remains to be further investigated.

**Trial registration:**

Name of registry: Clinical Trials Clinicaltrials.gov registration number: NCT02425462 Registration date: 2015-04-24. Registration timing: prospective.

## Background

Endometriosis is a chronic disease that affects approximately 10% of all women in reproductive age and up to 50% of infertile women [1, 2]. Current knowledge of various aspects of endometriosis is based on data primarily obtained from a Caucasian population; however there is scarce evidence among Asian women, where the prevalence of endometriosis appears to be increased compared to Caucasian women. Several studies reported an up to nine-fold increase in risk in Asian women as compared to the white female population [2–5].

Endometriosis is characterized by endometrial-like tissue outside the uterus. Conventionally, diagnosis of endometriosis required the combination of laparoscopic inspection of the pelvis with histological verification of endometrial glands, but this recommendation is not supported by robust evidence [6–8].

Guidelines recommend a non-invasive clinical diagnosis based on clinical symptoms and patients history [1, 6, 7]. However, as endometriosis symptoms appear non-specifically, the gap between the first symptoms and precise diagnosis ranges from four to 10 years [6]. In China, diagnosis is delayed on average even by 13 years [9]. Currently, there is no cure for endometriosis; rather, it is characterized by a progressive course with worsened symptoms if no appropriate therapy is applied [10]. In order to minimize disease progression, especially in women who wish to maintain their fertility, early diagnosis and proactive treatment is of big importance. Surgical intervention can substantially reduce pain and may increase fertility, but there is an increased risk of recurrence (40–50% at 5 years) and re-operation [11, 12]. Moreover, time to surgery can hinder appropriate treatment. Consequently, the current guidelines recommend a life-long, adapted management characterized by maximum medical treatment and prevention of repeated surgical procedures [7, 10]. In Asian countries, empirical medical treatment prior to or even without surgical intervention is widely practiced [9].

Classic endometriosis symptoms are chronic pelvic pain, dysmenorrhea, dyspareunia and infertility; furthermore, endometriosis can cause symptoms arising from other organs involved, such as dyschezia, tenesmus and dysuria and/or hematuria [13]. Quality of life studies reveal that endometriosis symptoms, especially chronic pelvic pain, can influence several aspects of a woman’s life, such as work, education, relationship, social support, especially with increasing severity of symptoms [10]. The impact of the disease on psychosocial parameters can lead to a significant reduction in health-related quality of life (HRQoL); thus, effective treatment of chronic pelvic pain is essential [10]. Nonsteroidal anti-inflammatory drugs, oral contraceptives and progestins are commonly considered first-line treatment for patients with endometriosis-associated pain [6, 10]. Dienogest is an oral progestin with a relatively short plasma half-life of approximately 9–10 h and high oral bioavailability of > 90%, offering unique pharmacologic benefits such as potent progestogenic effects that in turn result in pronounced endometrial lesion reduction. Furthermore, it is characterized by moderate suppression of gonadotropin secretion, anti-androgenic and anti-proliferative effects as well as good tolerability, rendering it an attractive long-term therapeutic approach [10, 14]. Importantly, dienogest demonstrated comparable efficacy with gonadotropin-releasing hormone (GnRH) agonists in reducing EAPP in clinical trials and is meanwhile approved for the treatment of endometriosis in 157 countries worldwide, including 15 in Asia [15, 16].

To date, the impact of dienogest treatment on quality of life has not been extensively studied in the real-life setting. Moreover, most of the clinical trials on dienogest enrolled only women with definite surgical diagnosis of endometriosis. This international non-interventional, non-controlled, multi-center, prospective cohort study (ENVISIOeN) aims to assess the effectiveness of dienogest (trade name VISANNE®, Bayer AG, Berlin, Germany) in improving HRQoL in clinically and surgically diagnosed Asian women with endometriosis under routine clinical practice. The interim analysis evaluated the pain domain of HRQoL as primary goal, as well as EAPP and safety as secondary goals after 6 months of follow up.

## Methods

### Study design

The study was carried out at 36 sites in Thailand (five centres), Indonesia (ten centres), Republic of Korea (twelve centres), Malaysia (four centres), Philippines (three centres) and Singapore (two centres) in accordance with the amended version of the Declaration of Helsinki (Oct 2013) and complied with Good Clinical Practice. For more information, see Clinicaltrials.gov (NCT02425462).

### Patients

The inclusion criteria were: Asian female ≥18 years old; clinical or surgical diagnosis of endometriosis; presence of EAPP; uninfluenced decision taken by the physician to newly prescribe dienogest; written informed consent. Women were excluded in case of participation in an investigational program with interventions outside of routine clinical practice; any contraindication listed in the local summary of product characteristics. All eligible patients were treated according to standard medical guidelines or usual care of the participating institute.

### Visits

The observation period of 24 months for each patient enrolled in this study consisted of the treatment phase and, in case of discontinuation of the treatment, the follow up period. Subjects were followed whether or not they remained on the treatment with dienogest. Information on the date and reasons for discontinuation were collected.

### Effectiveness variables

The primary endpoint was to assess the effectiveness of dienogest to change HRQoL with special attention to the dimension of pain. Patients were asked to complete the Endometriosis Health Profile-30 (EHP-30) at baseline and at the visits 6 months after start of treatment. The EHP-30 is a disease-specific, reliable and valid instrument to measure the effects of endometriosis on HRQoL, especially on physical, psychologic and social aspects, from the patients’ perspective, as described previously [17–19]. Secondary effectiveness endpoint variables comprised the assessment of changes in the other core as well as modular HRQoL domains and changes in the severity of EAPP from baseline to 6-months visits measured by patient-reported numeric rating scale (NRS) with a 4-week recall period at every visit.

### Safety variables

All TEAEs, including the ones leading to discontinuation were recorded at each visit throughout the study and summarized using the MedDRA coding system (not to be confused with the bleeding profile terminology). The bleeding profile was evaluated based on the following categories (definition within the figure): normal bleeding, irregular bleeding cycle, amenorrhea, intermenstrual bleeding/spotting. Further secondary endpoints analyzed within the scope of the interim analysis were continuation rate as well as patients’ and physicians’ satisfaction rate.

### Data sources and measurement

The treating physician collected historical data (demographic and clinical characteristics) from medical records if available and treatment-related data during visits that took place in routine practice.

### Bias

To circumvent the general bias susceptibility inherent to observational studies, e.g. selection bias, it ensured that the study population represented routine patient profiles treated with dienogest in Asia. The patients were selected only based on inclusion and exclusion criteria in a consecutive manner.

### Study size

The calculation of the sample size focused on the pain dimension in the EHP-30 score. Assumptions for the sample size have been based on earlier studies that collected information on quality of life in patients with endometriosis who have been treated daily with a progestin [20]. For this planned study similar results were expected. With a sample size of 696 subjects the 2-sided 99% confidence interval was expected to extend at most nominal +/− 2.25 in the score (which corresponds to +/− 4.5% of the expected mean change) with a probability of 95%. With a drop-out rate of about 20% it was advisable to include at least 870 subjects into this study.

In terms of safety this study was sufficiently large to capture uncommon adverse events. With the planned sample size at least one adverse event with a relative frequency of 0.3% was expected to be observed with a probability of 90%.

### Statistical analysis

Effectiveness analyses were conducted on the efficacy analysis set (EFF) that included all patients with evaluable EHP-30 questionnaire at baseline and at least one evaluable EHP-30 questionnaire post-baseline between week 12 and 36 after start of treatment. Measurements after discontinuation of treatment were excluded. Safety analyses were performed on the full analysis set (FAS) and comprised all patients that took at least one dose of dienogest.

The HRQoL parameters were assessed as described previously. The score ranged between 0 (best health status) and 100 (worst health status) [18]. For EAPP measured by patient-reported NRS, the patients were asked to circle one number in a range between 0, referring to “absence of pain” and 10, corresponding to “unbearable pain”. Changes in the severity of EAPP from baseline visit to 6-months visit, measured by patient-reported NRS at every visit were evaluated overall and separately for subgroups stratified by baseline severity of EAPP, use of rescue medication, prior treatment and method of diagnosis. The patient numbers for the conducted analyses vary due to variable numbers of complete and evaluable questionnaires, especially for analyses of mean changes.

The analysis was primarily of explorative and descriptive nature. All variables were analyzed descriptively with appropriate statistical methods. SAS release 9.4 (SAS Institute, Cary, NC, USA) was used for data analysis. Continuous variables were described by visit (baseline and 6-months) and as mean change from baseline to 6 months for each item of the questionnaire, if applicable. For change from baseline in EHP-30 scores descriptive statistics as well as confidence intervals have been provided. Frequency distributions of patients with improvement, deterioration or no change in EHP-30 and EAPP scores were summarized. Missing data was not replaced and was given as “missing” in the tables. All adverse events were presented within incidence tables as preferred terms by system organ class. The descriptive analysis of adverse events was the basis to assess tolerability and safety of dienogest in the study population. The analyses described were performed on TEAEs. Non TEAEs were tabulated without further stratification. Partially missing TEAE onset dates were imputed following a worst case approach. The earliest possible date was used (as described above), but in case the AE onset could be before or after start of dienogest, TEAE onset was imputed by the first day of dienogest treatment.

## Results

### Patient disposition and characteristics

Overall, a total of 895 patients were enrolled at 36 sites from 04/2015 to 08/2016. The FAS comprised 865 patients who were eligible, gave their informed consent and took at least one dose of dienogest. Furthermore, 510 patients with evaluable primary outcome were included into the EFF. The mean ± SD duration of treatment with end of observation was 14.1 ± 7.5 months (*n* = 97 evaluable patients; EFF). Leading reasons for end of observation were patient lost to follow-up (*n* = 37/97; 38.1%) and regular end of study (*n* = 35/97; 36.1%).

Baseline characteristics are summarized in Table 1. The most common symptoms of endometriosis were dysmenorrhea (*n* = 684/865; 79.1%) and chronic pelvic pain (*n* = 279/865; 32.3%). While endometriosis was diagnosed within 1 year before initial visit in the majority of the patients (*n* = 644/864; 74.5%), only less than half of the women reported onset of first symptoms within this time span (*n* = 402/865; 46.2%). Moreover, 29.9% of women (*n* = 259/865) documented appearance of first symptoms between one and 5 years before and 23.0% (*n* = 199/865) more than 5 years before. In fact, 24.6% of patients diagnosed within 1 year before initial visit experienced their first symptoms 1–5 years earlier and 12.4% of patients even > 5 years before, amounting to approximately 37% of patients with delayed diagnosis of endometriosis. The time period from appearance of first symptoms to first diagnosis tended to be shorter in surgically diagnosed patients than in patients with clinical only diagnosis (mean, 21.3 ± 43.9 months vs. 29 ± 44.8 months, respectively). The majority of women (*n* = 449/514; 87.4%) underwent prior surgery to treat endometriosis-associated pain. The proportion of women with pain recurrence despite surgical treatment was 27.4% (*n* = 123/449) and the mean ± SD duration until pain recurrence after surgery was 19.3 ± 21.0 months (duration was evaluable for 114 women). A total of 179 women received previous hormonal treatment, with 56.4% of those women (*n* = 101/179) suffering from pain recurrence after a mean ± SD time of 9.9 ± 13.9 months (evaluable for 97 women). Pain medications were given to 89 patients (of 514 with prior endometriosis treatment; 17.3%), predominantly 2–3 days per week (*n* = 50/89; 56.2%), with a mean ± SD pain recurrence after 11.9 ± 37.2 days (evaluable for 79 patients) after or during pain therapy.

**Table 1:** Baseline characteristics (to be placed after line 240)

Parameter		
Demography	n	years, mean ± SD
Age at registration	865	34.4 ± 7.6
History of endometriosis (*n* = 865)	n	% women
*Onset of first symptoms*^*a*^		
< 1 year ago	402	46.5
Between 1 and 5 years ago	259	29.9
> 5 years ago	199	23.0
Missing	5	0.6
*Most common symptoms of endometriosis*^*b*^		
Dysmenorrhea	684	79.1
Chronic pelvic pain	279	32.3
Dyspareunia	51	5.9
*Timepoint of first diagnosis*^*a*^		
< 1 year ago	644	74.5
Between 1 and 5 years ago	135	15.6
> 5 years ago	85	9.8
Missing	1	0.1
*Methods of diagnosis*		
Surgical diagnosis	616	71.2
Clinical diagnosis only	247	28.6
Missing	2	0.2
*Endometriosis lesions (n = 768 evaluable patients)*		
Single	417	54.3
Multi	351	45.7
*Endometriosis localization*^2^		
Ovary	640	88.4
Pelvic organ	282	39.0
Extra pelvic	21	2.9
r-ASRM stage of endometriosis (*n* = 246)^c^	n	% women
Stage I (minimal)	17	6.9
Stage II (mild)	20	8.1
Stage III (moderate)	86	35.0
Stage IV (severe)	126	51.2
Most common previous diseases (n = 141)^2^	n	% women
Uterine leiomyoma	69	48.9
Endometrial polyp	36	25.5
Ovarian cysts	26	18.4
Pelvic inflammatory disease	20	14.2
Most common concomitant diseases (*n* = 146)^2^	n	% women
Adenomyosis	46	31.5
Uterine leiomyoma	39	26.7
Anemia	18	12.3
Prior endometriosis treatment (*n* = 864 evaluable patients)	n	% women
Patients with prior treatment	514	59.5
Patients without prior treatment	350	40.5
Type of treatment (*n* = 514)	n	% women
Surgery	449	87.4
Hormonal treatment	179	34.8
Pain therapy	89	17.3

^*a*^Partially missing dates were imputed by the earliest possible time point: In case that only the day was missing, the date was imputed as the first day of the month. In case that the day and the month were missing, i.e. only the year was available the day and month was imputed by January 1st. ^2^Multiple answers possible. ^**c**^Assessed during surgical treatment; 3 patients underwent repeated surgery

### Effectiveness variables

#### Primary effectiveness variable

**Fig. 1:**
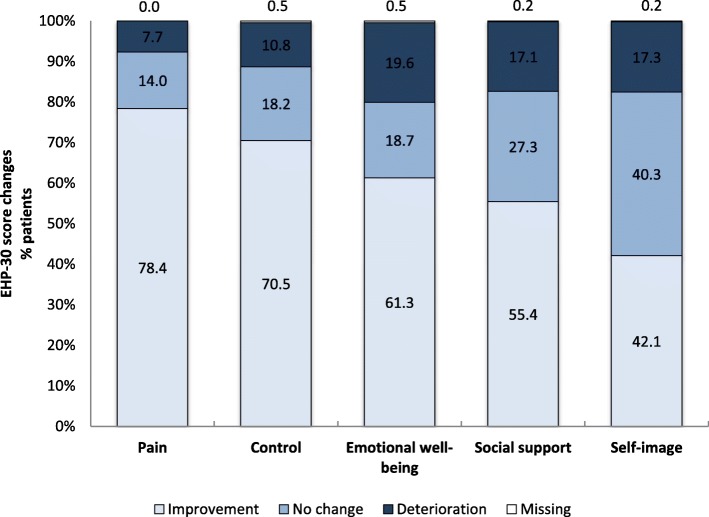
Assessment of (%) quality of life changes in EHP-30 core domains at 6 months (*n* = 444). Positive change indicates deterioration. Negative change indicates improvement

Table 2 summarizes the EHP-30 score distribution in the domains of the core questionnaire at baseline visit and 6-months visit, as well as the changes from baseline. The mean time ± SD point of measurement was 177.3 ± 22.1 days (n total = 444). Overall, the HRQoL nominally improved with regard to all reported domains at the 6-months visit compared to the baseline visit. Treatment with dienogest elicited a mean change ± SD of − 28.4 ± 27.3 (pain; 95%-CI: -31;-25.9), followed by mean ± SD changes of − 23.9 ± 27.9 (control; 95%-CI: -26.5;-21.3), − 14.7 ± 26.6 (emotional well-being; 95%-CI: -17.2;-12.3), − 12.9 ± 26.3 (social support; 95%-CI: -15.3;-10.4) and − 7.7 ± 25.6 (self-image; 95%-CI: -10.1;-5.3). Basically, dienogest treatment had the greatest impact on the dimensions of pain and control (Fig. 1). The proportion of patients with improvement of the pain score after 6 months was 78.4% (*n* = 348), whereas 14.0% (*n* = 62) showed no change and 7.7% (n = 34) reported deterioration. In the control domain, 70.5% (*n* = 313) of women experienced an improvement, whereas 18.2% (*n* = 81) and 10.8% (*n* = 48/444) had no change or deterioration, respectively. In the domains of emotional well-being, social support and self-image, 61.3% (*n* = 272), 55.4% (*n* = 256) and 42.1% (*n* = 187) of patients reported improvement, respectively.

**Table 2:** EHP-30 core scores and changes from baseline (to be placed after line 259)

Dimension	Timepoint	N	mean	Std	Q1	Median	Q3	Min	Max	Nmiss	CI 95%	CI 95%
Pain	Baseline	510	35.65	27.57	9.09	34.09	54.55	0.00	100.00	0	–	
	6 months	444	7.98	13.20	0.00	0.00	12.50	0.00	65.91	0	–	
	Change from baseline	444	−28.44	27.33	−47.73	−25.00	− 4.55	−100	36.36	0	−30.99	− 25.89
Control and powerlessness	Baseline	509	34.39	27.94	8.33	33.33	54.17	0.00	100.00	1	–	
	6 months	443	10.92	15.65	0.00	4.17	16.67	0.00	87.50	1	–	
	Change from baseline	442	−23.86	27.89	−41.67	−16.67	0.00	−100.00	50.00	2	−26.47	−21.25
Emotional well-being	Baseline	508	31.85	25.42	8.33	29.17	50.00	0.00	100.00	2	–	
	6 months	443	17.26	20.62	0.00	8.33	29.17	0.00	91.67	1	–	
	Change from baseline	442	−14.73	26.55	−33.33	−8.33	0.00	−100.00	79.17	2	−17.22	−12.25
Social support	Baseline	509	26.98	25.21	0.00	25.00	43.75	0.00	100.00	1	–	
	6 months	443	14.25	19.95	0.00	0.00	25.00	0.00	87.50	1	–	
	Change from baseline	443	−12.85	26.32	−31.25	−6.25	0.00	−100.00	87.50	1	−15.31	−10.39
Self-image	Baseline	509	19.97	24.06	0.00	8.33	33.33	0.00	100.00	1	–	
	6 months	443	11.87	19.38	0.00	0.00	16.67	0.00	91.67	1	–	
	Change from baseline	443	−7.67	25.58	−16.67	0.00	0.00	−100.00	91.67	1	−10.06	−5.29

#### Secondary effectiveness variables

Baseline and 6-months visit EHP-30 scores as well as changes within the modular questionnaire are summarized in Table 3. There, the domain of work was most changed upon dienogest therapy. The mean relative study day ± SD for the EHP-30 modular questionnaire was 177.5 ± 21.9 (*n* = 440). In the domain of work, the mean change ± SD was − 21.8 ± 25.3. Mean changes ± SD in other domains were as follows: − 19.5 ± 29.8 (relationship with children), − 11.4 ± 25.2 (sexual intercourse), − 7.2 ± 22.2 (treatment) and − 8.6 ± 21.7 (infertility).

**Table 3:** EHP-30 modular scores and changes from baseline (to be placed after line 267)

Dimension	Timepoint	N	mean	Std	Q1	Median	Q3	Min	Max	Nmiss	CI 95%	CI 95%
Work	Baseline	447	29.41	27.02	5.00	25.00	50.00	0.00	100.00	8	–	–
	6 months	398	7.01	13.05	0.00	0.00	10.00	0.00	70.00	5	–	–
	Change from baseline	366	−21.76	25.31	−40.00	−15.00	0.00	−90.00	40.00	76	−24.36	− 19.16
Relationship with children	Baseline	176	24.57	29.76	0.00	0.00	50.00	0.00	100.00	25	–	–
	6 months	174	5.24	13.45	0.00	0.00	0.00	0.00	75.00	25	–	–
	Change from baseline	130	−19.52	29.82	−50.00	0.00	0.00	−100.00	50.00	312	−24.69	−14.35
Sexual intercourse	Baseline	325	26.34	25.50	0.00	20.00	45.00	0.00	100.00	75	–	–
	6 months	278	14.93	20.82	0.00	2.50	25.00	0.00	95.00	34	–	–
	Change from baseline	238	−11.42	25.23	−25.00	−5.00	0.00	−90.00	75.00	204	−14.64	−8.20
Treatment	Baseline	361	19.25	22.37	0.00	16.67	33.33	0.00	100.00	27	–	–
	6 months	421	10.83	16.00	0.00	0.00	16.67	0.00	83.33	6	–	–
	Change from baseline	307	−7.19	22.18	−16.67	0.00	0.00	−91.67	50.00	135	−9.68	−4.70
Infertility	Baseline	303	27.97	28.47	0.00	18.75	50.00	0.00	100.00	29	–	–
	6 months	290	19.53	24.23	0.00	12.5	31.25	0.00	100.00	19	–	–
	Change from baseline	224	−8.62	21.74	−18.75	0.00	0.00	−100.00	56.25	218	−11.48	−5.76

**Fig. 2:**
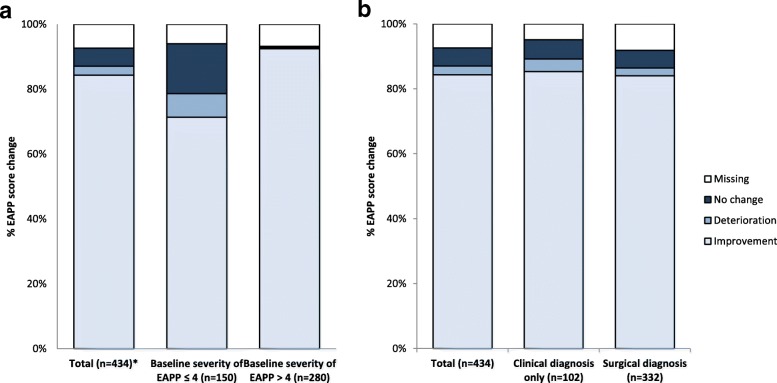
Evaluation of changes (%) in endometriosis-associated pelvic pain (EAPP) at 6 months (n = 434) according to EAPP baseline severity (**a**) and type of diagnosis (**b**). The changes have been assessed by numeric rating scale in a 4-week recall period. Positive change indicates deterioration. Negative change indicates improvement. *Change was not evaluable for four patients due to missing baseline severity of EAPP for three patients and one lost to follow-up

With regard to the EAPP assessment, the mean relative study day ± SD was 175 ± 23.4 (*n* = 434). The total study population reported a mean change ± SD of − 4.5 ± 3.0 (Table 4; *n* = 402). Remarkably, 84.3% (*n* = 366) of the total patient population described an improvement of EAPP with dienogest therapy, whereas 5.5% (*n* = 24) and 2.8% (*n* = 12) reported no change or even deterioration, respectively (Fig. 2). In line with the previous findings, baseline severity of EAPP had influenced patients’ pain reduction, as 92.5% (259/280) of women with EAPP baseline severity > 4 vs. 71.3% (*n* = 107/150) of women with EAPP baseline severity ≤4 experienced an amelioration of EAPP (Fig. 2a). Patients with EAPP baseline severity > 4 nominally had a greater benefit (mean change ±SD: − 6.2 ± 2.1; *n* = 261) compared to women with baseline severity ≤4 (mean change ±SD: − 1.4 ± 1.7; *n* = 141; Table 4). The type of diagnosis had no obvious influence on the effectiveness of dienogest to alleviate EAPP. The mean changes ± SD of − 4.3 ± 2.9 (*n* = 305) and − 5.1 ± 3.2 (*n* = 97) for surgically and clinically only diagnosed patients, respectively, were comparable to the mean change of the total population (Table 4). This was reflected by similar improvement rates of 84.0% (*n* = 279/332) in surgically diagnosed patients vs. 85.3% (*n* = 87/102) in clinically only diagnosed patients (Fig. 2b). Patients who took rescue medication at baseline showed a tendency towards an improved benefit from dienogest, as the mean change ± SD of − 5.6 ± 3.3 (*n* = 42) and consequent improvement rate of 93.0% (*n* = 40/43) marginally exceeded the mean change of the total population. Finally, the previous surgical or hormonal treatment did not affect the extent of EAPP change following dienogest administration (Table 4).

**Table 4:** EAPP scores and changes from baseline stratified by groups (to be placed after line 289)

Category	Stratification	Timepoint	N	mean	Std	Q1	Median	Q3	Min	Max	Nmiss
Total		Baseline	505	5.48	2.75	3.00	6.00	8.00	0.00	10.00	5
		6 months	405	0.97	1.43	0.00	0.00	2.00	0.00	7.00	29
		Change from baseline	402	−4.50	2.99	−7.00	−5.00	−2.00	−10.00	4.00	32
Method of diagnosis	Surgical diagnosis^a^	Baseline	388	5.30	2.75	3.00	6.00	7.00	0.00	10.00	3
		6 months	306	0.96	1.35	0.00	0.00	2.00	0.00	7.00	26
		Change from baseline	305	−4.31	2.92	−7.00	−4.00	−2.00	−10.00	4.00	27
	Clinical only diagnosis	Baseline	117	6.05	2.71	4.00	6.00	8.00	0.00	10.00	2
		6 months	99	1.01	1.66	0.00	0.00	1.00	0.00	6.00	3
		Change from baseline	97	−5.09	3.16	−8.00	−5.00	−3.00	−10.00	4.00	5
Baseline severity of EAPP	Baseline severity of EAPP ≤4	Baseline	176	2.28	1.19	1.00	2.50	3.00	0.00	4.00	0
		6 months	141	0.81	1.29	0.00	0.00	1.00	0.00	6.00	9
		Change from baseline	141	−1.44	1.66	−3.00	−1.00	−1.00	−4.00	4.00	9
	Baseline severity of EAPP > 4	Baseline	329	7.19	1.57	6.00	7.00	8.00	5.00	10.00	0
		6 months	261	1.05	1.48	0.00	0.00	2.00	0.00	7.00	19
		Change from baseline	261	−6.16	2.12	−8.00	−6.00	−5.00	−10.00	1.00	19
*Previous endometriosis treatment*	Previous surgical or hormonal treatment	Baseline	313	5.24	2.69	3.00	6.00	7.00	0.00	10.00	0
		6 months	242	0.93	1.40	0.00	0.00	1.00	0.00	7.00	19
		Change from baseline	242	−4.35	2.83	−7.00	−4.00	−2.00	−10.00	3.00	19
	No previous surgical or hormonal treatment	Baseline	191	5.88	2.82	3.00	6.00	8.00	0.00	10.00	5
		6 months	163	1.04	1.48	0.00	0.00	2.00	0.00	6.00	10
		Change from baseline	160	−4.74	3.21	−7.00	−5.00	−2.00	−10.00	4.00	13
Use of rescue medication	Use of rescue medication	Baseline	52	6.65	2.72	5.00	8.00	8.50	0.00	10.00	0
		6 months	42	1.29	1.86	0.00.	0.00	2.00	0.00	6.00	1
		Change from baseline	42	−5.55	3.31	−8.00	−6.00	−4.00	−10.00	4.00	1
	No use of rescue medication	Baseline	452	5.33	2.72	3.00	6.00	7.50	0.00	10.00	5
		6 months	362	0.94	1.37	0.00	0.00	1.00	0.00	7.00	28
		Change from baseline	359	−4.37	2.93	−7.00	−4.00	−2.00	−10.00	3.00	31

^a^Including patients with surgical diagnosis only and surgical+clinical diagnosis. *EAPP* endometriosis-associated pelvic pain

**Fig. 3:**
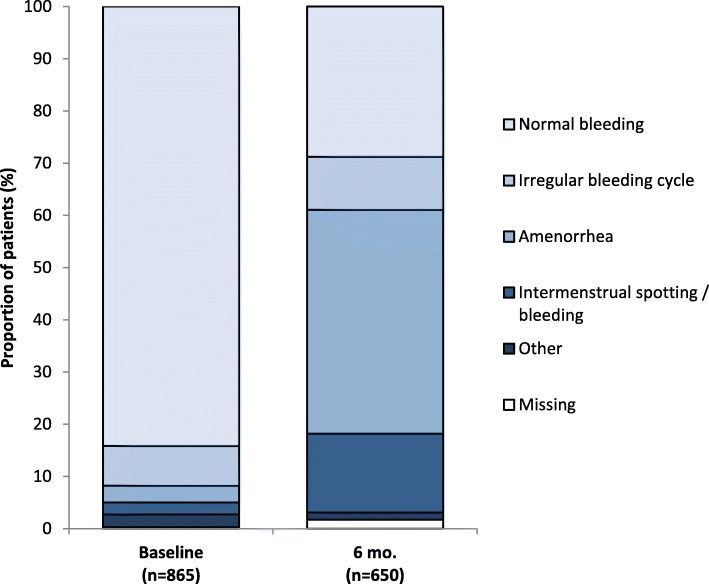
Changes (%) in the bleeding pattern from baseline to 6-months (6 mo.) visits. The bleeding patterns were defined as follows: 1) Normal bleeding: Regular bleeding with normal flow and duration. 2) Irregular bleeding cycle: Bleeding cycle less than 21 days or more than 35 days. 3) Amenorrhea: No menstruation during last 90 days. 4) Intermenstrual spotting / bleeding: Irregular episodes of bleeding, often light and short, occurring between otherwise fairly normal menstrual periods. Normal bleeding decreased from 84.2% at baseline to 28.8% after 6 months. Irregular bleeding cycle increased from 7.6 to 10.2%, amenorrhea from 3.2 to 42.9%, intermenstrual bleeding/spotting from 2.3 to 15.1%.

With regard to the bleeding profile of the patients, normal bleeding was decreased from 84.2% (*n* = 728/865) to 28.8% (*n* = 187/865) after 6 months, whereas amenorrhea increased from 3.2% (*n* = 28/865) to 42.9% (n = 279/865), as displayed in Fig. 3.

A large proportion of patients (66%; *n* = 322/488) and physicians (66.8%; *n* = 326/488) were satisfied with the therapy. Perceivable overall symptom improvement was reported by 52.1% (*n* = 254/488) of cases. Finally, the majority of patients continued treatment with dienogest (88.1%; *n* = 430/488) after 6 months.

### Safety variables

In total, there were nine serious adverse events (SAEs) reported in eight patients (Table 5). Thereof, anaemia (2/9 events) represented the most common. In eight cases the reason for seriousness was “Hospitalization necessary or prolonged”, in the remaining case “Important medical event”. All SAEs were recovered or resolved. Drug-related TEAEs were documented for 272 of 865 patients (31.5%), with amenorrhea (5.9%), metrorrhagia (5.1%) and vaginal haemorrhage 4.5%) representing the most common. A minority of patients (*n* = 45/865; 5.2%) discontinued dienogest therapy due to drug-related TEAEs. Abnormal uterine bleeding (preferred terms: vaginal haemorrhage, menorrhagia and metrorrhagia) was the most prominent reason for discontinuation (0.9%; *n* = 8/865). TEAEs irrespective of causality were documented in 35.5% of patients (*n* = 307/865) and were predominantly of mild-to-moderate intensity. Most common TEAEs are presented in Table 6.

**Table 5:** All documented serious adverse events (to be placed after line 309)

Serious Adverse Event (MedDRA PT)	Events		Patients	
	n	%	n	%
Anaemia	2	22.22	2	0.23
Bartholin’s abscess	1	11.11	1	0.12
Leptospirosis	1	11.11	1	0.12
Peritonitis	1	11.11	1	0.12
Dysmenorrhoea^a^	1	11.11	1	0.12
Menorrhagia^a^	1	11.11	1	0.12
Ovarian cyst	1	11.11	1	0.12
Not coded yet^b^	1	11.11	1	0.12
Any PT	–	–	8	0.96
Total	9	100.00	865	100.00

^a^Drug-related serious adverse events.^b^not coded until cut-off date. *FAS* full analysis set, *MedDRA* Medical Dictionary for Regulatory Activities; *PT* preferred term

**Table 6:** Incidences and rates of most frequent adverse events (i.e., at least 2% of patients), FAS (to be placed after line 309)

Adverse Event (MedDRA PT)	Events		Patients	
	n	%	n	%
Amenorrhoea	69	12.08	58	6.71
Metrorrhagia	69	12.08	51	5.90
Vaginal haemorrhage	41	7.18	39	4.51
Menstruation irregular	28	4.90	25	2.89
Headache	27	4.73	27	3.12
Acne	20	3.50	20	2.31
Alopecia	18	3.15	18	2.08
Weight increased	17	2.98	17	1.97
Total^a^	571	100.00	865	100.00

^a^67 events were not coded until cut-off date. *FAS* full analysis set, *MedDRA* Medical Dictionary for Regulatory Activities; *PT* preferred term. Order of illustration is based on the frequency of events

## Discussion

The debilitating, chronic and relapsing nature of endometriosis points to an unmet need for effective treatment approaches [10]. A study on women’s pain revealed that 40% of women with endometriosis are dissatisfied with their current treatment, supportive of the fundamental necessity to improve the long-term management of debilitating pain and consequently, of HRQoL [21]. Importantly, endometriosis represents an economic and social burden on both families and society. Delayed diagnosis, high hospitalization, surgical procedures and impaired HRQoL are outcomes from the variable and chronic presentation of endometriosis symptoms [10]. Interestingly, several studies point to a higher prevalence of endometriosis in Asian women [3–5]. Particularly in low resource settings, the management of endometriosis should ideally be incorporated into the primary health care of women. Therefore, first-line medical therapy should focus on feasible drugs with favorable efficacy as well as long-term safety and tolerability profile. Dienogest has been extensively studied in four key European regulatory Phase II and III trials, as well as in numerous clinical trial programs performed in Europe and Asia [10, 15, 22]. More precisely, 2 mg dienogest once daily demonstrated significant efficacy for lesion reduction and reduction in pain intensity as well as convincing safety and tolerability data [15, 22]. Two large trials with treatment duration of 52 and 65 weeks concluded that dienogest is suitable for an effective long-term management of endometriosis, as it was associated with sustained reduction of pain intensity, predictable adverse events and in turn low discontinuation rates. Notably, beneficial effects on quality of life could be observed for up to 1 year [10].

Accordingly, our data suggest that dienogest might represent an option in the treatment of endometriosis in Asian women in the real-life setting, particularly in the improvement of HRQoL. As such, nominal improvements throughout all domains of the EHP-30 questionnaire after 6 months compared to baseline were observed. The most striking change was documented in the domain of pain, where an improvement in 78.4% of patients after 6 months was documented. Importantly, the changes in the other core scores were in line with changes in the domains of the modular questionnaire, underscoring the relevance to relieve pain for the improvement of all aspects of HRQoL. As some aspects of QoL such as social support and self-image might be affected through other psychosocial factors than EAPP, these domains appeared to be less influenced by dienogest. With regard to the EAPP, dienogest therapy was associated with a change of pain after 6 months, and an improvement rate of 84.3%. Women with higher EAPP score > 4 at baseline visit described greater improvement (92.5%) at 6-months visit, in contrast to 71.3% improvement in women with low baseline severity of EAPP ≤4.

According to recent guidelines, endometriosis should be classified with questions about pelvic pain and infertility in low resource settings in order to prescribe the most successful treatment possible prior to or even without definite surgical/histological confirmation [23]. Thus, early medical treatment without histological confirmation is endorsed [7]. A relevant proportion (28.6%) of women enrolled in our study was exclusively clinically diagnosed, and, moreover, our baseline data indicate a delayed diagnosis after appearance of the first endometriosis symptoms in 37% of women. Moreover, women with surgical diagnosis appeared to be somewhat earlier diagnosed than clinically only diagnosed women. Interestingly, dienogest showed comparable effectiveness to reduce EAPP in women with surgical and clinical diagnosis in our study. This finding might be of significance for the clinical practice especially in low resource settings where patients do not have access to surgical facilities or need to delay surgery for medical or personal reasons.

The bleeding profile was affected by dienogest therapy, with decreased normal bleeding (28.8%) and increased amenorrhea (42.9%) after 6 months. Commonly, abnormal uterine bleeding is a well-known adverse event upon long-term treatment with progestins. A pooled analysis of clinical trials revealed that amenorrhea increased from < 5 to 30% upon extended treatment duration, whereas undesirable patterns such as abnormal, frequent or prolonged bleeding decreased [10, 14–16, 24]. This highlights the importance of informing the patients about the potential adverse events in order to sustain adherence to therapy. As the overall discontinuation rate within this study was low (10.7%), with no discontinuation due to amenorrhea, and eight discontinuations due to abnormal uterine bleeding, we assume that the changes in the bleeding patterns were tolerated well by the patients. In accordance with data from other studies, we observed mild-to-moderate drug-related TEAEs with amenorrhea and metrorrhagia being the most common. One of the two patients with drug-related SAEs (menorrhagia) had reported adenomyosis as concomitant disease which could be considered as causal factor of the SAE as well. Nevertheless, both drug-related SAEs as well as the majority of all drug-related AEs were recovered and the associated discontinuation rate was low (5.2%), with overall seven discontinuations due to abnormal bleeding, one among them for metrorrhagia and menorrhagia, respectively, and six discontinuations due to vaginal haemorrhage. Taken together, safety of dienogest in Asian women with endometriosis is comparable to the existent observations in other countries [14–16].

This international non-interventional study design allows observing dienogest use among different Asian countries under real-world clinical settings for the first time. Therefore, all decisions in terms of diagnostic procedures and management of the disease are fully dependent on mutual agreement between the patient and the treating physician, without interference by a sponsor. As discussed above, there is an unmet need to improve the impaired quality of life of women with endometriosis, highlighting the clinical relevance of the study. A potential limitation of this study was the low number of evaluable patients included in the EFF, resulting from a lower feasibility of regular participation for many patients in the real-world setting. The local conditions at the participating sites possibly hampered the women to comply with the scheduled time points. Furthermore, there were certainly differences between the healthcare systems of the participant countries, in particular in terms of diagnosis and treatment. The major limitation of this study however is that within a single arm design lacking appropriate controls, the effects on HRQoL and safety cannot be fully attributed to the dienogest therapy. Other unknown factors might be underestimated and therefore, effectiveness and safety of dienogest can only be interpreted to a limited extent and requires further investigation.

## Conclusion

In conclusion, our data indicated that therapy with dienogest could improve HRQoL and change the perception of EAPP in Asian women suffering from debilitating endometriosis symptoms. As no new safety signals were observed and a good satisfaction rate and compliance was achieved, dienogest might be an interesting therapeutic option for the long-term management of endometriosis among Asian women.

## Abbreviations

EAPP: Endometriosis-associated pelvic pain
EFF: Efficacy analysis set
EHP-30: Endometriosis Health Profile-30
FAS: Full analysis set
GnRH: Gonadotropin-releasing hormone
HRQoL: Health-related quality of life
NRS: Numeric rating scale
SAE: Serious adverse event.
TEAE: Treatment-emergent adverse event
